# Maine

**Published:** 1896-04

**Authors:** 


*Maine.* State Veterinarian Bailey, of Maine, recently tested a herd of forty cattle in that State with tuberculin, seventeen of which responded, and on being destroyed proved tuberculous. Ten hogs on the same farm, fed with skimmed milk (the product of the farm being used for butter-making) were found tuberculous on being killed for food-purposes.

